# Water Impact on Superhydrophobic Surface: One Hydrophilic Spot Morphing and Controlling Droplet Rebounce

**DOI:** 10.3390/biomimetics10050319

**Published:** 2025-05-15

**Authors:** Jiali Guo, Haoran Zhao, Ching-Wen Lou, Ting Dong

**Affiliations:** 1 College of Textile and Clothing, Qingdao University, 308 Ningxia Road, Qingdao 266071, China; jlguo040506@163.com (J.G.); mingxiaosama@outlook.com (H.Z.); cwlou@ctust.edu.tw (C.-W.L.); 2 Advanced Medical Care and Protection Technology Research Center, Qingdao University, 308 Ningxia Road, Qingdao 266071, China; 3 Advanced Medical Care and Protection Technology Research Center, Department of Fiber and Composite Materials, Feng Chia University, Taichung City 407102, Taiwan; 4 School of Chinese Medicine, China Medical University, Taichung City 404333, Taiwan

**Keywords:** surface wettability engineering, superhydrophobicity, contact line, droplet impact, droplet bouncing regulation

## Abstract

Motion control of droplets undergoing collisions with solid surface is required in a number of technological and industrial situations. Droplet dynamics after lifting off is often unpredictable, leading to a major problem in many technologies that droplets move in uncontrolled and potentially undesirable ways. Herein, this work shows that well-designed surface chemistry can produce an accurate control of force transmission to impinging droplets, permitting precise controlled droplet rebounce. The non-wetting surfaces (superhydrophobic), which mimics the water-repellent mechanism of lotus leaves via micro-to-nanoscale hierarchical morphology, with patterned “defect” of extreme wettability (hydrophilic), are synthesized by photolithography using only one inexpensive fluorine-free reagent (methyltrichlorosilane). The contact line of impinging droplet during flatting and receding is free to move on the superhydrophobic region and pinned as it meets with the hydrophilic defect, which introduces a net surface tension force allowing patterned droplet deposition, controlled droplet splitting, and directed droplet rebound. The work also achieves controlled vertical rebound of impinging droplets on inclined surfaces by controlling defect’s size, impact position, and impact velocity. This research demonstrates pinning forces as a general strategy to attain sophisticated droplet motions, which opens an avenue in future explorations, such as matter transportation, energy transformation, and object actuation.

## 1. Introduction

Engineering surfaces that control the movement of liquid droplet is of importance to a wide range of applications including self-cleaning [1,2], anti-icing [3,4], anti-fogging [5,6], electricity generation [7,8], heat transfer [9,10], and droplet-based microfluidics [11,12]. A drop striking a non-wetting surface will spread out to maximum diameter and then recoil to rebound in the vertical direction [13]. The dynamics of droplet impact is altered when such surface has a morphology. This controlled droplet impact is critical to many technologies.

Droplet impact on hydrophobic surfaces decorated with submillimeter superhydrophobic textures presents pancake bouncing or petal bouncing, which allowed rapid drop detachment [14,15]. Superhydrophobic ridges could break drop impact symmetry and reduce the contact time by modified droplet deformation near the miniature features [16,17]. The springboard effect was generated for a droplet impacting an elastic superhydrophobic surface, which led to a twofold reduction in contact time [18]. Droplet impacting on a curved surface displayed asymmetric bouncing with a ~40% contact time reduction when the curvature was on the order of the droplet diameter [19]. Also, drops impacting on a convex surface with a dome shape could rapidly bounce off by evolving into an annulus shape where the inner and external rims had a high retracting velocity [20]. Oblique drop impact on superhydrophobic surface with two-tier roughness could bounce off the surface rapidly in an elongated shape, allowing a 10%∼30% reduction of contact time [21]. An ellipsoidal drop impacted on a superhydrophobic surface is demonstrated to reduce the contact time and suppress the bounce magnitude by breaking the symmetry [22]. Moreover, translational motion of an impacting droplet was converted to gyration, with a maximum rotational speed exceeding 7300 revolutions per minute, through heterogeneous surface wettability regulation [23]. Numerous researches of droplet impact are focused on minimizing contact time [24]. In contrast, drop dynamics after the restitution process, which is often unpredictable, attracts little attention. The result is that droplets can move in uncontrolled and potentially undesirable ways after impacting the surface, which is a major problem in many technologies [25]. One approach to droplet rebounce control is engineering nonuniform textures (i.e., roughness gradients) on the surface. Droplets impacting on a rough surface with a wettability gradient was shown to rebound obliquely or migrate following the wettability gradient due to the unbalanced interfacial forces created by wetting difference across the droplets [26]. However, the result is that droplets did not always move following the direction of decreasing static CA due to the energy barrier caused by the Cassie-to-Wenzel transition [27]. The wetting symmetry of a droplet can be broken at high temperature by creating two concurrent thermal states (Leidenfrost and contact-boiling) on a surface with structural roughness gradient, which also induce a preferential motion of a droplet towards the region with a higher heat transfer co-efficiency [28,29].

In contrast to complex surface roughness design, this work shows that the dynamic behavior of impacting droplets was precisely controlled by well-designed surface chemistry. The non-wetting surfaces with patterned “defect” of extreme wettability was synthesized in a simple and straightforward way. The “defect” of extreme wettability on non-wetting surfaces introduced a net surface tension force to impinging droplets, allowing patterned droplet deposition, controlled droplet splitting, and directed droplet rebound. The work also shows how defect’s size, impact position, and impact velocity alter the dynamic behavior of impacting droplets, achieving controlled vertical rebound of impinging droplets on inclined surfaces. This research shows attaining sophisticated droplet motions by pinning forces originated from chemically engineered surfaces, which opens an avenue in future explorations, such as matter transportation, energy transformation, and object actuation.

## 2. Materials and Methods

### 2.1. Materials

Deionized water was purified using a Millipore Milli-Q system that involves reverse osmosis, ion exchange, and filtration steps (1018 Ω/cm). Silanes (methyltrichlorosilane) obtained from Gelest were used without purification to further treat the substrates. Silicon wafers (100 mm diameter, ~500 μm thickness) were used as the substrates to create well-designed surface chemistry. Shipley S1813 photoresist and Microposit MF-321 developer were obtained from Microchem Corp (Westborough, MA, USA). Photolithography masks were printed on polyester film via CAD/Art Services, Inc (Bandon, Oregon, USA). Ethanol and anhydrous toluene were purchased from Aldrich (Shanghai, China).

### 2.2. Surfaces Preparation

The non-wetting surfaces (superhydrophobic) were synthesized by the following fabrication procedures. Wafers were cleaned using a Harrick plasma cleaner operated at 30 W for 20 min. The chamber was evacuated to less than 150 mTorr and blended with oxygen to maintain a pressure of 300 mTorr during the plasma cleaning. The cleaned wafers were placed in a scintillation vial filled with 10 mL anhydrous toluene. 20 μL MTCS (Methyltrichlorosilane) was added in by a syringe. The system was subsequently exposed to a highly humidified environment (RH = 60~80%) controlled by a glove bag for 1~3 min. The vial was then caped and kept at room temperature for 24 h. After the reaction, the modified wafers were cleaned with toluene, ethanol, and water. The obtained surfaces were non-wetting surfaces.

**Figure 1:**
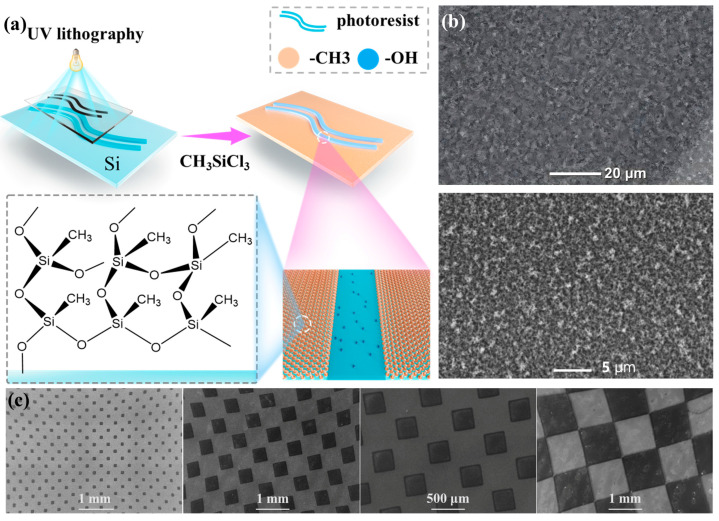
(**a**) Schematic showing the procedure to prepare patterned surface; (**b**) SEM of the non-wetting regime; (**c**) SEM micrographs showing alternating superhydrophobic and hydrophilic square grid chessboard from 50 to 1000 μm in width.

To patterning “defect” of extreme wettability (hydrophilic) on the non-wetting surfaces, photolithography was carried out on silicon wafers. As indicated in Figure 1a, an oxygen plasma-cleaned silicon wafer was spin-coated with photoresist for 60 s at 2000 rpm and then baked at 120 °C for 3 min to remove solvents. UV exposure was carried out using a 500 W (365 nm) OAI UV lamp. After exposure to UV for 16 s through a particular mask, the wafer was submerged in developer for around 60 s to remove resist from the exposed regions, rinsed with deionized water for 1 min, and then further cleaned with oxygen plasma for 20 min. This procedure produced wafers with resist-coated patterns on Si/SiO_2_ background. The wafer with patterned photoresist was then treated using the above development procedures for non-wetting surfaces. This area-selective reaction yielded non-wetting surfaces with patterned photoresist. The remaining photoresist was removed by rinsing with acetone and water (Milli-Q). After resist removal, the wafer section contained a superhydrophobic background and hydrophilic patterns.

Surface’s contact angles (WCA) were measured using a Rame–Hart telescopic goniometer with a Gilmont syringe and a 24-gauge flat-tipped needle. Dynamic advancing and receding angles were recorded while the probe fluid, Milli-Q water, was added to and withdrawn from the drop, respectively. Each WCA was decided by the five repeats.

Field emission scanning electron microscope (SEM, Zeiss Sigma500, Carl Zeiss AG, Oberkochen, Germany) was used to observe the structure of the surface. The working distance for SEM were 3–5 mm, at an energy beam of 10 keV and 10 μA.

### 2.3. Experimental Setup

Our experiments involve releasing a water droplet (radius R_0_ = 1.23 mm) onto a superhydrophobic surface patterned with hydrophilic circle (“defect”, radius R_defect_ = 0.75–2.00 mm) and filming the bounce dynamics with high-speed cameras (Figure S1). Five water droplet impact positions were designed. The maximum extension of the droplet is theoretically calculated by $R_{\max}\approx R_{0}We^{0.25}/4$ [30]. Position P1 is the droplet hitting on the center of the hydrophilic circle (the distance between impact point and center of hydrophilic circle L_dis_ = 0). P2 is the droplet hitting on the edge of the hydrophilic circle (L_dis_ = R_defect_). Position P3 is the droplet impacting and spreading to just cover the hydrophilic circle (L_dis_ = R_max_ − R_defect)_. On position P4, the droplet impacts and spreads to just touch the edge of the hydrophilic circle (L_dis_ = R_max_ + R_defect_). For position P5, droplet impacting and spreading does not touch the hydrophilic circle (L_dis_ > R_max_ + R_defect_). The impact distance from the tip of the needle to the surface of the sample was H = 3–15 cm, with impact velocity v = 0.77–1.71 m/s, We = 9.8–49.0.

## 3. Results and Discussion

Well-designed surface chemistry is used to produce an accurate control of force transmission to impinging droplets, permitting precise controlled droplet rebounce. The non-wetting surfaces are patterned with “defect” of extreme wettability (hydrophilic) by photolithography. The non-wetting regime (superhydrophobic) are synthesized by a simple and straightforward way using only one inexpensive fluorine-free reagent (methyltrichlorosilane) without complex or costly fabrication procedures. We emphasize that this procedure was carried out in a lab with access to an oxygen plasma cleaner, spin coater, and UV lamp. Photolithography was carried out on the silicon wafer using photoresist spin coating, illumination, and development procedures [31,32]. After area-selective development yielding resist-coated patterns and Si/SiO_2_ background (θ_A_/θ_R_ = ~56°/~24°), the wafer section was exposed to a 20 min O_2_ plasma treatment (Harrick PDC-001). This treatment cleans the background and oxidizes/ablates the outer few nanometers of the ~1.3 μm thick resist. This surface was then treated with CH_3_SiCl_3_ for introducing silicone nanofilaments that impart superhydrophobicity. After resist removal, the wafer section contained a superhydrophobic background with hydrophilic patterns. As shown in Figure 1b, the non-wetting regime are covered with a layer of condensed micro-size nanofibers/particles, which mimics the water-repellent mechanism of lotus leaves via micro-to-nanoscale hierarchical morphology. The hydrophilic patterns were determined by what the applied photolithography masks were and could be made in any shape. Figure 1c demonstrated the resolution of this photolithography/chemistry procedure with SEM micrographs that showed alternating superhydrophobic and hydrophilic rings and a square grid chessboard from 50 to 1000 μm in width.

**Figure 2:**
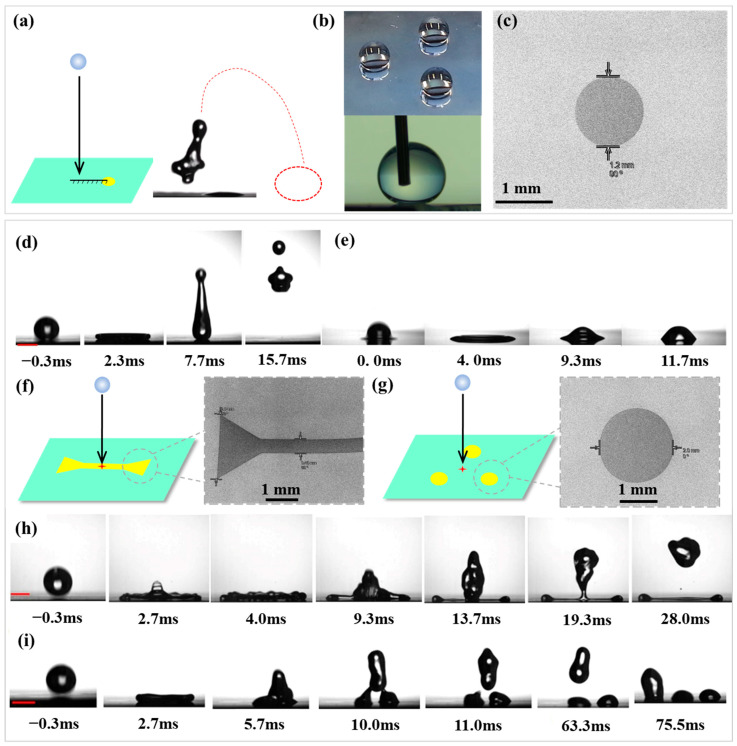
(**a**) Schematic representation of droplet impact dynamics; (**b**) Contact angle configurations on superhydrophobic/hydrophilic surfaces (upper: droplet centered on hydrophilic circular domain; lower: droplet on superhydrophobic region); (**c**) SEM of the hydrophilic circle on superhydrophobic surfaces; Photographs of a water droplet dropped from a height of 5 cm to different surface. (**d**) non-patterned superhydrophobic surface (treated with CH_3_SiCl_3_); (**e**) non-patterned silicon hydrophilic surface (untreated Si wafer). (**f**) superhydrophobic surface patterned with two hydrophilic triangles (2 × 2 × 2 mm) connecting by a line (6 × 0.5 mm) (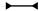) and one end of this pattern’s SEM image; (**g**) superhydrophobic surfaces containing three hydrophilic circles (radius = 2 mm) (**⸫**) and one circle of this pattern’s SEM image; Photographs of a water droplet dropped from a height of 5 cm to superhydrophobic surface containing different hydrophilic patterns. (**h**) superhydrophobic surface patterned with two hydrophilic triangles connecting by a line (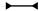); (**i**) superhydrophobic surfaces containing three hydrophilic circles (**⸫**).

As shown in Figure 2a, the non-wetting surfaces containing one hydrophilic cycle are designed to produce an accurate control of force transmission to impinging droplets. Figure 2b and Figure S1 illustrates one piece of such patterned surface with images of water droplets confined by three hydrophilic circles (radius = 1.2 mm, one circle of this pattern was image by SEM in Figure 2c) on the superhydrophobic surfaces. The background surface exhibits contact angles of θ_A_/θ_R_ = 172°/164°, and the hydrophilic circles show contact angles of θ_A_/θ_R_ = ~56°/~24°. The disparity of advancing contact angles between the two areas defined the shape of water droplets acting as three eggs of a table-like construction which supports a piece of PET film (Figure S1).

For Weber numbers $\mathrm{We}=2\rho v^{2}R_{0}/\gamma$, where ρ, R_0_, v and γ are the droplet density, initial radius, impact speed, and surface tension, respectively, the maximum extension of droplet is theoretically calculated by $R_{\max}\approx R_{0}We^{0.25}/4$ [30]. Based on this, five water droplet impact positions were designed as shown in Figure S2. Position P1 is the droplet hitting on the center of the hydrophilic circle (the distance between impact point and center of hydrophilic circle L_dis_ = 0). P2 is the droplet hitting on the edge of the hydrophilic circle (L_dis_ = R_defect_). Position P3 is the droplet impacting and spreading to just cover the hydrophilic circle (L_dis_ = R_max_ − R_defect)_. On position P4, the droplet impacts and spreads to just touch the edge of the hydrophilic circle (L_dis_ = R_max_ + R_defect_). For position P5, droplet impacting and spreading does not touch the hydrophilic circle (L_dis_ > R_max_ + R_defect_). The impact distance from the tip of the needle to the surface of the sample was H = 3–15 cm, with impact velocity v = 0.77–1.71 m/s, We = 9.8–49.0. The outcome of droplet impacting is affected by receding contact angle at the contact line [33]. For droplet impacting onto superhydrophobic surface, the contact line advances and then recoils (rebounds) (Figure 2d), while the contact line on hydrophilic surface is pinned without recoil (deposition) (Figure 2e). The droplet impacting can be tailored with controlled ending by chemically engineering a surface with wetting patterns. Figure 2f–i illustrates this with two examples. In Figure 2d, a water droplet impacting onto a non-patterned superhydrophobic surface (treated with CH_3_SiCl_3_) spreads to a uniform film, retracts, and then lifts off (see Video S1). For a water droplet impacting onto a superhydrophobic surface patterned with two hydrophilic triangles (2 × 2 × 2 mm) connecting by a line (about 6 × 0.5 mm) (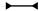, one end of this pattern was imaged by SEM in Figure 2f) in Figure 2h, a centra assisted spread is promoted. The droplet impacted spreads fast along the pattern with the outward flow generating cups at the ends. The contact line on the hydrophilic pattern gets pinned during recoil; on the superhydrophobic region, the contact line continues to recede until the outward flow impacts the inward flow, leaving a hydrophilic region defined water pattern on the surface with the rest of droplet take-off (see Video S2). Another example in Figure 2i shows a superhydrophobic surfaces containing three hydrophilic circles (radius = 2 mm) (⸫, one circle of this pattern was imaged by SEM in Figure 2g). The droplet impact on the centra of three hydrophilic circles. The droplet spreads and recedes, but the contact line on the hydrophilic circles gets pinned during recoil, which tailors the water droplet into three drops after an impact (see Video S3).

**Figure 3:**
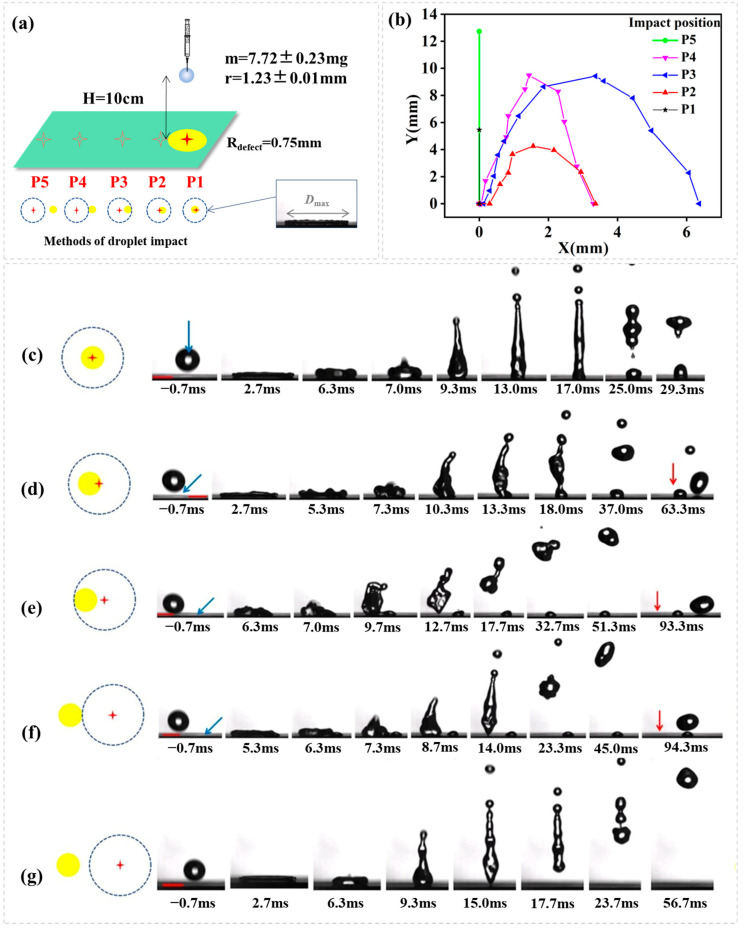
(**a**) Schematic representation of droplet impact dynamics; (**b**) Droplet rebouncing trajectory. A plot of lateral distance (X) vs. vertical distance (Y) for droplets impacting on five different positions. The image sequence showing a water droplet impacting (H = 10 cm, We = 32.67) onto superhydrophobic surface containing a hydrophilic circle (R_defect_ = 0.75 mm). (**c**–**g**) indicates different impact position as the right shows, the red cross means the impact point, the yellow circle means the hydrophilic defect, and the blue dotted line means the theoretical maximum spread of the droplet, the blue arrow means the position of hydrophilic defect, the red arrow means the position of impact point, the red scale bar is 2.00 mm.

The emphasis of this article is controlling the droplet rebounce dynamics using the superhydrophobic surfaces including a hydrophilic defect. In the hydrophilic defect containing surfaces, the disparity of apparent contact angle from the hydrophilic to superhydrophobic region develops a net surface tension force during the receding of droplet impact in one direction potentially allowing directed droplet rebounce. The first experiment shows how the impact position relative to a hydrophilic defect alters droplet rebounce trajectory (Figure 3a). Figure 3b illustrates droplet rebounce trajectory with a plot of lateral distance (X) vs. vertical distance (Y) for droplets impacting on five different positions. Figure 3c–g shows the image sequence as seen from the side.

For a water droplet impacting with defect size R_defect_ = 0.75 mm, impact distance H = 10 cm (We = 32.67), we see from Figure 3 that the outcomes of droplet impacting on different positions are quite different. Droplet impacting and spreading that does not touch the hydrophilic defect (P5, L_dis_ > R_max_ + R_defect_) behaves as if it is in contact with an isotropic superhydrophobic surface, on which the water droplet after an impact spread to a nearly uniform film, retracts symmetrically and lifts off that is orthogonal to the surface. In another case that the water droplet exactly right hits the hydrophilic defect (P1, L_dis_ = 0), the contact line is free to move on the superhydrophobic region until it recedes reaching the hydrophilic circle, at which point the contact line is pinned. This draw the droplet into two fragments in the end: small lobe leaves on the hydrophilic defect and the rest of the drop flies up vertically to a height that is much lower than that in the usual rebounce. For droplet hits which spreads to touch the hydrophilic defect on one side (e.g., P2, P3, P4), a net surface tension force develops during receding, allowing directed droplet rebounce. As shown in Figure 3c–g, the symmetry of the droplet is broken during receding. The contact line on the superhydrophobic region is free to move and is pinned on the hydrophilic defect that is located on one side. A net surface tension starts to occur as the contact line recedes to contact with the hydrophilic defect, which recomposes the droplet from the symmetric disk to a shape (e.g., gun) that is ready to launch in a direction that is not orthogonal to the surface. It is important to note that the distance between impact point and the hydrophilic defect is response for different droplet rebounce trajectory. As we see from Figure 3b, droplet impacting and spreading to just cover the hydrophilic circle (P3, L_dis_ = R_max_ − R_defect_) allows the longest lateral leap.

**Figure 4:**
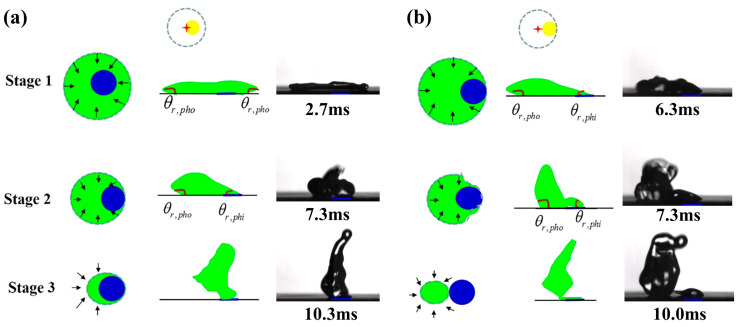
Typical recoil process of droplet impacting on (**a**) P2 (L_dis_ = R_defect_) and (**b**) P3 (L_dis_ = R_max_ − R_defect_) portraying a possible mechanism for impact position effect. Stage 1 to stage 3 means three stages of receding, *θ_r,pho_* means the receding contact angle on the superhydrophobic area, *θ_r,phi_* means the receding contact angle on the hydrophilic defect. The black arrows in stage 1-3 represent the moving direction of the droplet’s receding contact line. The blue cycles in stage 1-3 represent the hydrophilic regime.

A central mechanism for impact position effect is the position of the hydrophilic defect, which starts to meet with the receding contact line. Figure 4 portrays the typical recoil process of droplet impacting on P2 (L_dis_ = R_defect_) and P3 (L_dis_ = R_max_ − R_defect_). In the case of P2, the droplet after reaching its maximum spread continues to recede on the superhydrophobic region with high receding contact angle (θ*_r_* = θ*_r,pho_*) until the receding contact line reaches the hydrophilic defect, at which point the liquid gets pinned on the hydrophilic area due to the disparity of receding contact angle from the superhydrophobic to the hydrophilic region. A net surface tension force develops vectoring the droplet up to the instant of detachment. For the case of P3, a net surface tension force develops at the instant of the droplet’s maximum lateral spread and continues to the instant of detachment. As a result, the droplet is directed to a longer lateral leap than that of in the former case. Video S4 demonstrates the process of droplet impacting on P3.

**Figure 5:**
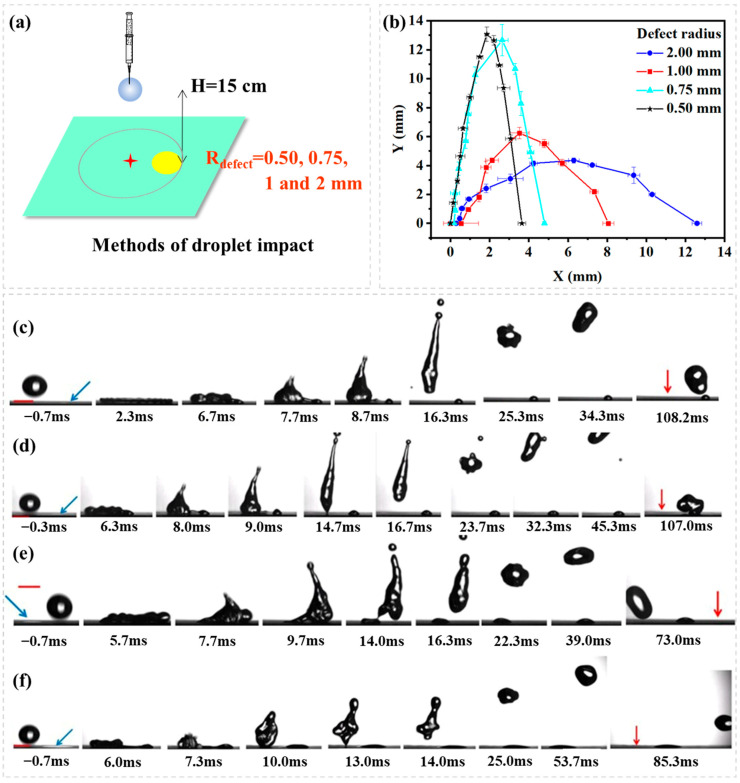
(**a**) Schematic representation of droplet impact dynamics; (**b**) A plot of lateral distance (X) vs. vertical distance (Y) for droplets impacting onto different sizes of hydrophilic circles (R_defect_ = 0.05, 0.75, 1.00, 2.00 mm) from an impact position where the droplet after an impact spreads to just cover the hydrophilic circle (P3, L_dis_ = R_max_ − R_defect_). The image sequence shows a water droplet impacting (H = 15 cm, We = 49.00) onto a superhydrophobic surface containing a hydrophilic circle of different sizes. (**c**) R_defect_ = 0.50 mm; (**d**) R_defect_ = 0.75 mm; (**e**) R_defect_ = 1.00 mm; (**f**) R_defect_ = 2.00 mm; the blue arrow means the position of hydrophilic defect, the red arrow means the position of impact point, the red scale bar is 2.00 mm.

The second experiment shows the effect of hydrophilic defect size on droplet rebouncing dynamics Figure 5a. Figure 5b illustrates droplet rebounce trajectory with a plot of lateral distance (X) vs. vertical distance (Y) for droplets impacting onto different sizes of the hydrophilic circle (R_defect_ = 0.05, 0.75, 1.00, 2.00 mm) from an impact position where the droplet after am impact spreads to just cover the hydrophilic circle (P3, L_dis_ = R_max_ − R_defect_). Figure 5c–f shows the image sequence as seen from the side. The receding contact line meets with the defect as the receding begins, which continuously peels off (de-wetting) the hydrophilic circle up to the instant of detachment (rebound), at which point the droplet is re-shaped to—launch parabolically. The de-wetting contact line on the hydrophilic circle is increased with the increase of the hydrophilic circle’s size. This leads to a general trend that the lateral momentum of droplet rebounce is increased with the increase of hydrophilic defect’s size and that the longitudinal momentum of droplet rebounce is decreased with the increase of the hydrophilic defect’s size.

**Figure 6:**
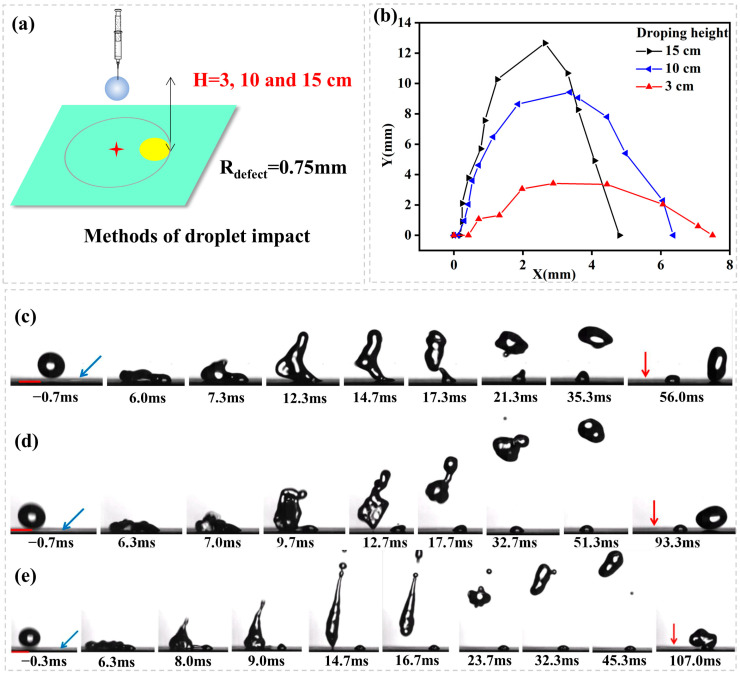
(**a**) Schematic representation of droplet impact dynamics; (**b**) A plot of lateral distance (X) vs. vertical distance (Y) for droplets impacting from different height (H = 3, 10, 15 cm, We = 9.80, 32.67, 49.00). The droplet hits on position P3 and spreads to cover a hydrophilic defect (R_defect_ = 0.75 mm). The image sequence showing a water droplet impacting from (**c**) H = 3 cm, We = 9.80, (**d**) H = 10 cm, We = 32.67, (**e**) H = 15 cm, We = 49.00 onto a superhydrophobic surface containing a hydrophilic circle of R_defect_ = 0.75 mm and hits on position P3. In (**c**–**e**) the blue arrows indicate the locations of hydrophilic defects and the red arrows indicate the initial droplet impact locations on the surface.

The third experiment shows the influence of impact height on the directed rebounce (Figure 6a). Figure 6b illustrates droplet rebounce trajectory with a plot of lateral distance (X) vs. vertical distance (Y) for droplets impacting from different heights (H = 3, 10, 15 cm, *We* = 9.80, 32.67, 49.00, respectively). The droplet hits on position P3 and spreads to cover a hydrophilic defect (R_defect_ = 0.75 mm). Figure 5c–e shows the image sequence as seen from the side. It is noted from Figure 5 that the decrease of impact height decreases the longitudinal momentum of droplet rebounce because of the decrease of initial impact kinetic energy. However, it is important to note that the lateral momentum of droplet rebounce is increased with the decrease of impact height. The reason can be identified from Figure 6c–e. As the droplet impacts the surface, its kinetic energy is directed to the lateral direction, flattening the droplet with the kinetic energy converting into surface energy. Once all of the kinetic energy has been converted to surface energy of the flattened droplet, the reverse surface-to-kinetic energy conversion initiates, resulting in droplet retraction. The decrease of impact height decreases the initial kinetic energy and thus leads to the reduction of retraction speed (which is theoretically expressed as $\sqrt{2\gamma /\rho h}$, where γ is the liquid-air surface tension, *ρ* is the liquid density, and *h* is the thickness of the flattened droplet). This finally leads to the droplet flying up before totally peeled off from the hydrophilic defect. Therefore, a lateral force continues to work until the main droplet is divorced from the pining contact line by capillary break-up.

**Figure 7:**
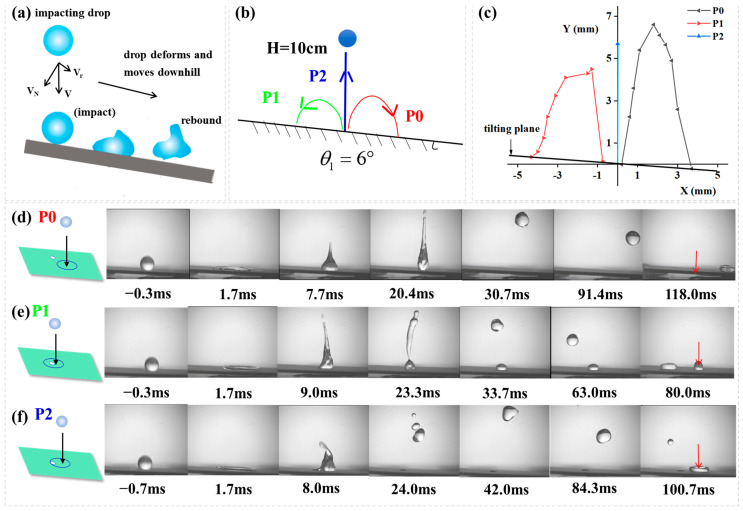
(**a**) Illustration of droplet impact dynamics on an inclined surface; (**b**) Schematic representation of droplet impact dynamics (H = 10 cm) on an inclined surface (θ *=* 6°), containing a hydrophilic circle (R_defect_ = 0.75 mm) (**c**) Droplet rebouncing trajectory. A plot of lateral distance (X) vs. vertical distance (Y) for droplets impacting on three different positions. The image sequence showing a water droplet impacting from (**d**) P0, (**e**) P1, (**f**) P2. In (**d**–**f**) the red arrows indicate the initial droplet impact locations on the surface.

In addition to droplet motion on horizontal surfaces, the pinning forces were further exploited to achieve controlled vertical rebound of impinging droplets on inclined surfaces by controlling the defect’s size, impact position, and impact velocity. As indicated in Figure 7a, compared to droplet bouncing on a horizontal surface, the behavior on a tilted surface is significantly more complex [34]. When a droplet impacts a tilted superhydrophobic surface, its motion follows a dynamic and asymmetric process. The initial impact causes the droplet to spread rapidly, with greater expansion in the downhill direction due to the surface tilt. The droplet then quickly retracts because of the surface’s low adhesion, and under the influence of gravity’s downhill component, it typically rebounds downward or transitions directly into a rolling motion. If the impact energy is high (indicated by a large Weber number), the droplet may undergo several diminishing oblique bounces before rolling. If the kinetic energy is low or the liquid viscosity is high (high Ohnesorge number), the droplet accelerates rapidly into a rolling motion along the slope. Throughout this process, the droplet’s trajectory remains biased toward the downhill direction, and its speed changes in three stages, including deceleration upon impact, acceleration during retraction/rebound, and stabilization during rolling, before finally detaching from the surface in an almost straight path. Since the pinning forces can provide moments to the liquid droplet, water droplets impacting on a tilted surface should also be controlled. To test this idea, a water droplet was released from H = 10 cm and hit a superhydrophobic surface tilted at 6° containing a hydrophilic circle (R_defect_ = 0.75 mm), as indicated in Figure 7b. Figure 7c illustrates the rebound trajectories of droplets impacting different positions on the inclined surface, plotted as the relationship between horizontal distance (X) and vertical distance (Y). Figure 7d–f present side-view image sequences of the impact process. When impacting at position P0 (Figure 7d), the droplet did not spread sufficiently to reach the hydrophilic defect site, resulting in a normal downward rebound along the slope direction. In contrast, Figure 7e demonstrates the rebound process for droplet impacting at position P1, where the droplet spread far enough to reach the hydrophilic spot, and an upward-directed pinning force along the inclined surface propelled the droplet to ascend along the slope against gravitational forces. For the case in Figure 7f, where the droplet hit at position P2, the equilibrium between an inclination-parallel upward pinning force and gravitational components enabled vertical rebound of impacting droplets, exhibiting impact dynamics analogous to droplets colliding on horizontal surfaces. The complete impacting process at P2 is recorded in Video S5.

## 4. Conclusions

In summary, this study presented an investigation into controlling water micro-volumes through droplets impacting on superhydrophobic surfaces containing a well-designed hydrophilic defect. The contact line of droplet during receding is free to move on the superhydrophobic region and pinned as it meets with the hydrophilic defect, which introduces a net surface tension force allowing shaped droplet rebounce, including patterned droplet deposition, controlled droplet splitting, and directed droplet rebound. It is important to note that the distance between impact point and the hydrophilic defect is responsible for the different droplet rebounce trajectory. A central mechanism for impact position effect is the position of the hydrophilic defect, which starts to meet with the receding contact line. The result also demonstrates the capability to manipulate rebounce behavior by controlling defect size and impact velocity. The work also achieves controlled vertical rebound of impinging droplets on inclined surfaces by controlling defect’s size, impact position, and impact velocity. This research demonstrates pinning forces as a general strategy to attain sophisticated droplet motions, which opens an avenue in future explorations, such as matter transportation, energy transformation, and object actuation.

## Data Availability

Data are contained within the article.

## Supplementary Materials

The following supporting information can be downloaded at: https://www.mdpi.com/article/10.3390/biomimetics10050319/s1, Figure S1: A water droplet striking the superhydrophobic surface (indicated by green area) patterned with a hydrophilic circle (indicated by yellow area), P1 to P5 indicate five different impact position, the red cross means the impact point, and the blue dotted line means the theoretical maximum spread of the droplet; Figure S2: Water droplets on superhydrophobic surfaces containing three hydrophilic circles with a radius of 1 mm. (a) 5 μL, (b) 10 μL, (c) 15 μL water droplet confined by the three hydrophilic circles; (d) a piece of PET film supported by three water droplets exhibiting a table-like construction. Video S1: A water droplet impact onto a non-patterned superhydrophobic surface (treated with CH_3_SiCl_3_); Video S2: A water droplet dropped from a height of 5 cm to a superhydrophobic surface patterned with two hydrophilic triangles (2 × 2 × 2 mm) connecting by a line (6 × 0.5 mm) (); Video S3: A water droplet dropped from a height of 5 cm to a superhydrophobic surfaces containing three hydrophilic circles (radius = 2 mm) (**⸫**); Video S4: The process of droplet impacting on P3; Video S5: The complete impacting process at P2 on inclined surface.
