# Supplementary material for: Water Impact on Superhydrophobic Surface: One Hydrophilic Spot Morphing and Controlling Droplet Rebounce
Source: Biomimetics (Basel). 2025 May 15;10(5):319. doi: 10.3390/biomimetics10050319 (PMC12109196; doi:10.3390/biomimetics10050319)
Supplement: Supplementary file 1 [file biomimetics-10-00319-s001.zip › Supporting Information.pdf]

Title:

Water impact on superhydrophobic surface: one hydrophilic spot morphing and controlling droplet rebounce

Authors and affiliations:

Jiali Guo [a, b, 1], Haoran Zhao [a, 1], Ching-Wen Lou [a, b, c, d], Ting Dong [a, b, c, *]

[a] College of Textile and Clothing, Qingdao University, 308 Ningxia Road, Qingdao, P.R. China

[b] Advanced Medical Care and Protection Technology Research Center, Qingdao University, 308 Ningxia Road, Qingdao, P.R. China

[c] Advanced Medical Care and Protection Technology Research Center, Department of Fiber and Composite Materials, Feng Chia University, Taichung City 407102, Taiwan

[d] School of Chinese Medicine, China Medical University, Taichung City 404333, Taiwan

***Corresponding author:**

tingdong09@qdu.edu.cn (Ting Dong)

[1] These authors contributed equally to this work.

Present/permanent address:

College of Textile and Clothing, Qingdao University, #308, Ningxia Road, Qingdao, 266071, P.R. China

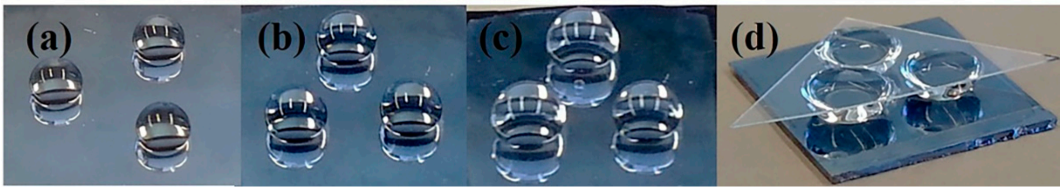

Figure S1. Water droplets on superhydrophobic surfaces containing three hydrophilic circles with radius of 1 mm. (a) 5 μL, (b) 10 μL, (c) 15 μL water droplet confined by the three hydrophilic circles; (d) a piece of PET film supported by three water droplets exhibiting a table-like construction.

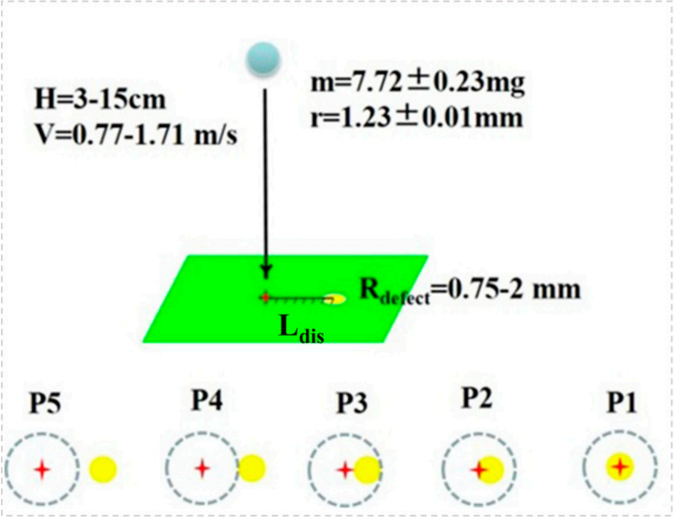

Figure S2. A water droplet striking the superhydrophobic surface (indicated by green area) patterned with a hydrophilic circle (indicated by yellow area), P1 to P5 indicate five different impact position, the red cross means the impact point and the blue dotted line means the theoretical maximum spread of droplet.
